# Evaluating the Social Media Performance of Hospitals in Spain: A Longitudinal and Comparative Study

**DOI:** 10.2196/jmir.6763

**Published:** 2017-05-23

**Authors:** Antonio Martinez-Millana, Carlos Fernandez-Llatas, Ignacio Basagoiti Bilbao, Manuel Traver Salcedo, Vicente Traver Salcedo

**Affiliations:** ^1^ ITACA Universitat Politècnica de València Valencia Spain; ^2^ Unidad Mixta de Reingeniería de Procesos Sociosanitarios (eRPSS) Instituto de Investigación Sanitaria Hospital Universitario y Politecnico La Fe Valencia Spain; ^3^ Hospital de Manises Valencia Spain

**Keywords:** public health, delivery of health care, Internet, social media, hospitals

## Abstract

**Background:**

Social media is changing the way in which citizens and health professionals communicate. Previous studies have assessed the use of Health 2.0 by hospitals, showing clear evidence of growth in recent years. In order to understand if this happens in Spain, it is necessary to assess the performance of health care institutions on the Internet social media using quantitative indicators.

**Objectives:**

The study aimed to analyze how hospitals in Spain perform on the Internet and social media networks by determining quantitative indicators in 3 different dimensions: presence, use, and impact and assess these indicators on the 3 most commonly used social media - Facebook, Twitter, YouTube. Further, we aimed to find out if there was a difference between private and public hospitals in their use of the aforementioned social networks.

**Methods:**

The evolution of presence, use, and impact metrics is studied over the period 2011- 2015. The population studied accounts for all the hospitals listed in the National Hospitals Catalog (NHC). The percentage of hospitals having Facebook, Twitter, and YouTube profiles has been used to show the presence and evolution of hospitals on social media during this time. Usage was assessed by analyzing the content published on each social network. Impact evaluation was measured by analyzing the trend of subscribers for each social network. Statistical analysis was performed using a lognormal transformation and also using a nonparametric distribution, with the aim of comparing *t* student and Wilcoxon independence tests for the observed variables.

**Results:**

From the 787 hospitals identified, 69.9% (550/787) had an institutional webpage and 34.2% (269/787) had at least one profile in one of the social networks (Facebook, Twitter, and YouTube) in December 2015. Hospitals’ Internet presence has increased by more than 450.0% (787/172) and social media presence has increased ten times since 2011. Twitter is the preferred social network for public hospitals, whereas private hospitals showed better performance on Facebook and YouTube. The two-sided Wilcoxon test and *t* student test at a CI of 95% show that the use of Twitter distribution is higher (*P*<.001) for private and public hospitals in Spain, whereas other variables show a nonsignificant different distribution.

**Conclusions:**

The Internet presence of Spanish hospitals is high; however, their presence on the 3 main social networks is still not as high compared to that of hospitals in the United States and Western Europe. Public hospitals are found to be more active on Twitter, whereas private hospitals show better performance on Facebook and YouTube. This study suggests that hospitals, both public and private, should devote more effort to and be more aware of social media, with a clear strategy as to how they can foment new relationships with patients and citizens.

## Introduction

The Spanish health care system is one of the best ranked in the world as far as patient safety [1], efficiency [2], and satisfaction [3] is concerned. The health sector in Spain represents 9.5% (118.9/1.252 USD trillion) of the gross domestic product (2.5% for private purposes and 7% for public services). The Public health care copes the majority of delivery of health services, but in a recent study it was shown that private hospitals performed 32% (1.12/3.50 million) of surgeries, responded to 21% (9.91/47.2 million) of emergencies, and took up 15% of outpatients’ referrals (11.83/78.9 million) [4]. Moreover, in a recent survey made on Spanish citizens looking for medical assistance, 60% said they preferred being assisted in a public hospital, whereas 28% preferred private hospitals [5]. This was because they considered public hospitals to have better technological installations, more capable physicians and nurses, and because public hospitals provided more and better information than private hospitals [5]. Therefore, information plays a big role in the way citizens make their choices about health management, and the need for information is increasing exponentially [6]. The need for accurate information is particularly critical when the information involves health, well-being, and disease, especially when Internet sources are winning the battle over traditional sources of information [7].

Internet health-related queries rose from 2010 [7-9], and they were mainly used to support a decision, such as looking for a second opinion or even purchasing drugs [10]. Even junior physicians consult information provided on the Internet to reinforce the diagnosis and treatment decisions they make on a daily basis [11]. One example of how people use Internet sources to assess their illness is Wikipedia [12,13], which hosts a large quantity of information about medical data [14], even comparable with commercial encyclopedias [15]. The easy access and the easy-to-understand development of health topics are turning Wikipedia into the first-choice Internet source to find brief and clear definition of a specific term, including health terminologies [14].

This scenario is defining a new paradigm in which health services’ consumers and procurers (patients and health professionals) share a new framework for information exchange [16]. The unstoppable advance of social media in medicine is now a reality, and it is pushing health professionals and hospitals to learn, start, and increase their use of social media as a communication channel. Business concepts are currently being studied to improve marketing strategies for hospitals [17] and to amplify health values and principles [18]. These developments reflect the manner in which health professionals are applying their knowledge and experience in terms of interacting with patients [19].

Hospitals cannot control the information in social media [20]; on the other hand, patient communities have taken the lead in allowing the sharing of medical experiences on social media [21], and some social media sites have empowered patients to provide personal ratings on their health care experiences [22]. The relationship between hospital social media activity and quality key performance indicators are still quite unexplored; however, it has become increasingly critical to find effective ways of communicating with the community outside clinical environments as traditional communication channels such as Web 1.0, electronic mail, and media campaigns are being replaced by new communication channels [16]. Three of the key indicators used previously are (1) the presence, defined as the rate of health care entities with a profile or page on a social network; (2) the use (or usage), defined as the number of posts with content published in a time window; and (3) the impact, defined as the capability of an entity to gain subscribers [23,24]. In this paper we present a 5-year longitudinal study on the use of webpages and social media among public and private hospitals in Spain to evaluate the aforementioned indicators. Our hypothesis is that public and private hospitals perform differently, as regards to the final target of each type of entity. Presence, use, and impact of social media profiles have been analyzed to determine how metrics have evolved over time and which direction they will take in the future, by comparing the performance of public and private hospitals over 5 years on the 3 main social networks: Facebook, Twitter, and YouTube. Finally, results are compared with previous publications in the United States and Western Europe. The statistical analysis of the data allows us to confirm that there is a statistically significant difference in the use of Twitter between private and public hospitals. Spain is progressing well in the adoption and use of social media, but our findings reinforce the need to promote new forms of communication by public hospitals in the era of social communication, by using innovative channels to reach a bigger audience.

## Methods

### Study Design

A longitudinal review of hospitals that have presence on 3 of the most popular social networks—Facebook, Twitter, and YouTube—was conducted. For each hospital, data about the use and user acceptation of the generated content was collected, as well as general information about the hospital (eg, public or private ownership).

### Data Collection

The studied cohort included all the hospitals listed in the “National Catalog of Public and Private Hospital Centers (NHC)” maintained by the Spanish Ministry of Health and Social Affairs [25]. The overall study cohort included 787 hospitals. Webpages (Web 1.0) and social media profiles were discovered using contact data, such as the name of the institution, address, and municipality on the Google search engine. The sites were validated by accessing the search resulting pages manually and verifying that the content corresponded to the appropriate hospital. Only institutional profiles were included in the study population. Personal, department, service, or unofficial profiles were not included. Hospital ownership (public or private) was obtained from the NHC. Retrieved social media profiles for each hospital were classified as belonging to 1 of 3 social media networks—Facebook, Twitter, or YouTube. In order to avoid the effect of stationary events (eg, winter or summer campaigns), the temporal window to retrieve data was fixed from January 2011 to December 2015. As in previous studies, these 3 social networks were selected because of their popularity and the possibility of accessing performance metrics. Data included whether hospitals had accounts on social networks (presence), their activity on those accounts (use), and how those activities were received by the intended audience (impact).

### Statistical Analysis

The study assessed 3 factors: presence, usage, and impact, similar to previous studies [23,26]. The percentage of hospitals having Facebook, Twitter, and YouTube profiles was used to indicate the presence of hospitals on social media. Usage was assessed by analyzing the content generated on each social network (eg, number of tweets and videos) over the period studied. Impact was measured by the number of subscribers for each social media account. These 3 factors are considered sufficient to evaluate the extent to which hospitals are present in social media, to assess their performance on social media in terms of communication (using number of posts), and whether users were consuming the disseminated content by subscribing to a particular account or channel. All these indicators were analyzed with respect to hospital ownership: public or private. For all the observed variables, we present the median, interquartile range, and kurtosis value. Goodness of fit to a normal distribution was evaluated by two techniques. First, according to [26], a lognormal transformation was used to approximate the skewed distribution of the variables to a normal distribution; a nonparametric distribution was used to obtain the raw probability density function. Both approximations were compared with raw data to assess reliability. Independence of public and private hospital results was assessed with the two-sided, *t* student test, and Wilcoxon test (CI of 95%) for each distribution. Statistical significance was considered for *P* values under .05. MATLAB statistics toolbox (version 2016R) was used to perform correlation, transformation, and independence tests [24].

## Results

### Presence Dimension

**Figure 1:**
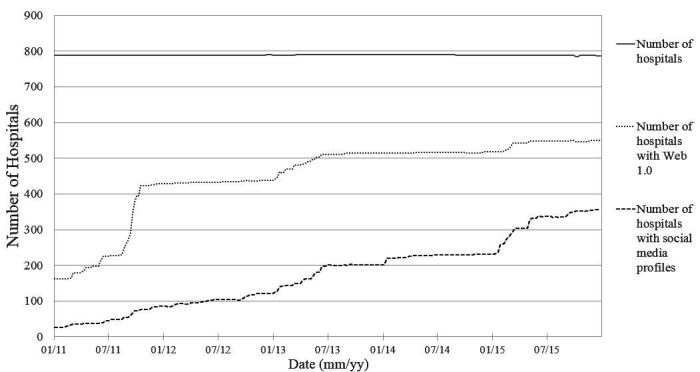
Evolution of Internet presence of Spanish hospitals in the period 2011-2015.

From a total of 787 hospitals identified in the NHC of the Ministry of Health and Social Affairs, 550 had an institutional webpage, and 269 of them had at least one profile in one of social networks considered in December 2015. Figure 1 shows the evolution of Internet presence among public and private hospitals from 2011 to 2015. Even though Internet presence increased by more than 450.0% (787/172) and social media presence increased ten times since 2011, there are still many hospitals without Web 1.0 and social media profiles. However, the correlation is strong between the evolution of hospitals with an institutional webpage (Web 1.0) and presence in social media, with a value coefficient of 0.949. In December 2015, from the total number of hospitals (787) only 69.9% (550) had an official Web 1.0 and only 34.2% (269) were present in any of the social networks considered. Taking into account only those hospitals with an Internet presence, up to 48% (264) of them had a profile page in at least one of the studied social networks.

Beyond new hospital openings and new profiles on social networks, variations in the number of hospitals (continuous line in Figure 1) are due to modifications and updates done in the NHC. Variations in the number of Web 1.0 pages and social media profiles are due to the corporative acquisitions of the owners of private hospitals and profile relabeling, resulting in a merger of social media profiles.

**Figure 2:**
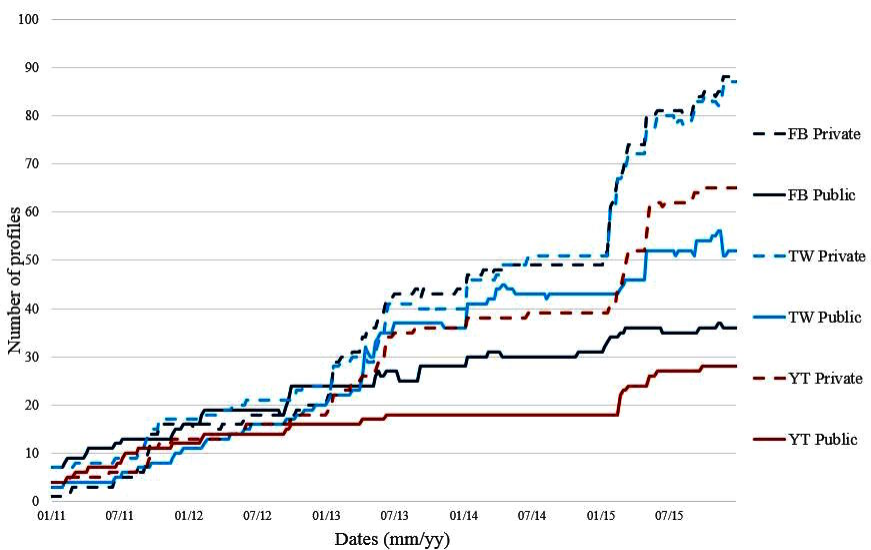
Evolution of social media profiles of public and private hospitals from 2011 to 2015. Facebook (FB), Twitter (TW), and YouTube (YT) and the type of hospital are represented using a private or public token (eg, FB Private stands for private hospital Facebook profiles).

The aggregated and comparative distribution of the presence of public versus private hospitals shows that public hospitals have less Internet presence than private hospitals, not only regarding Web 1.0 pages where we find 24% (186) versus 29% (283) but also in the use of social media to disseminate information on their activities, which accounts for 33% (262) versus 60% (419). Focusing on the presence (profiles in social media), Figure 2 shows the trend from 2011 to 2015. It shows that the creation of profiles is very similar (correlation coefficient=.971), but that private hospitals present a viral growth in two specific periods (first semesters of 2013 and 2015—plain transition means no variation and a step transition means a high transition).

Despite the fact that social media has a lower presence overall in public hospitals, there are more public than private hospitals using Twitter (44% vs 36%), whereas with Facebook and YouTube, it is the opposite (31% vs 36% and 24% vs 27%). With respect to YouTube, the presence percentage is similar for private and public hospitals.

Data of the historical evolution of social media profiles disaggregated by social media type and public or private hospitals (Figure 2) confirms the previous result, and even though there exists a strong correlation between the creations of social media profiles, a slightly different volume is observed depending on the social network and entity; public hospitals have fewer profiles considering absolute numbers.

**Figure 3:**
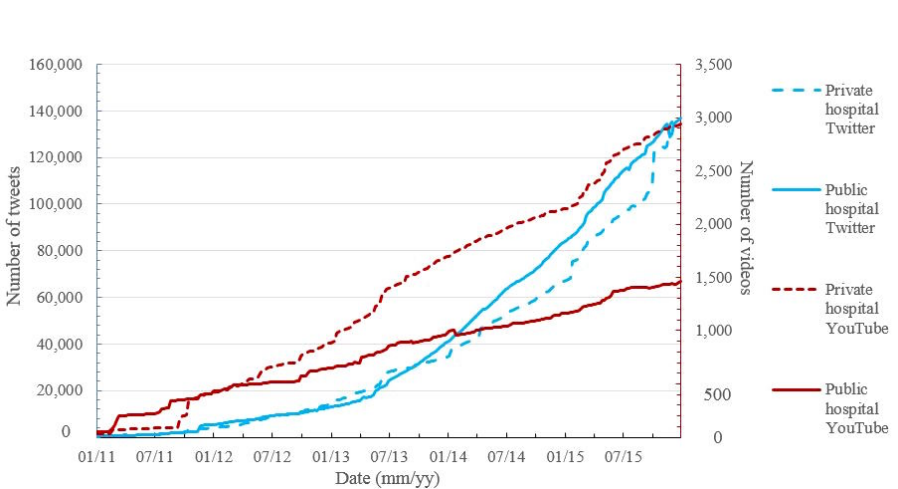
Evolution of use of Twitter (left y-axis) and YouTube (right y-axis) among private and public hospitals in the period 2011-2015.

### Use Dimension

Where analysis of content generation by hospitals in social media (Figure 3) is concerned, different behavior depending on social media and the type of entity is evident. Both public and private hospitals seem to have the same activity in Twitter, whereas private hospitals double the use of YouTube with respect to the public sector.

### Impact Dimension

**Figure 4:**
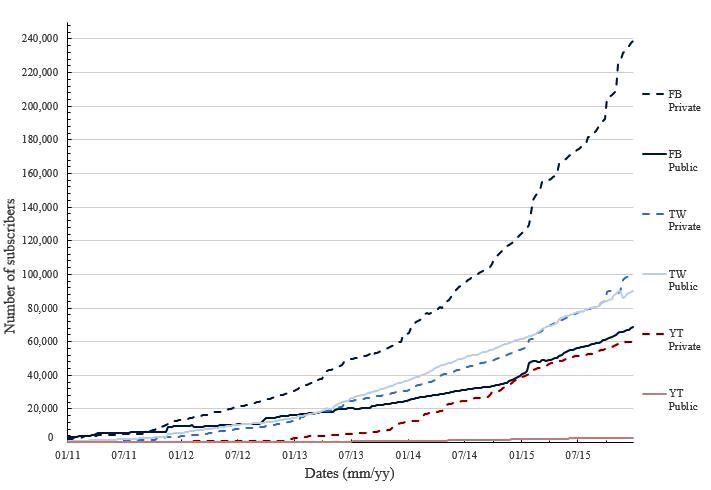
Impact evolution of private and public hospitals in social media. Facebook (FB), Twitter (TW), and YouTube (YT) and the type of hospital are represented using a private or public token (eg, FB Private stands for private hospital Facebook profiles).

The evolution in the number of subscribers for the studied social networks for both private (dotted line) and public (continuous line) hospitals is exponentially increasing (Figure 4). All the profiles show continuous incremental growth, similar to an exponential function. The coupled analysis of the subscribers shows that Facebook is the most popular network, followed by Twitter. YouTube is, by far, the social network with the lowest subscriptions. Analysis of ownership shows that subscriptions to Facebook private hospitals profiles are significantly higher than those for public hospitals. With respect to Twitter subscribers, public hospitals have almost the same number as private and show a very similar trend of growth over the years. This growth is proportional to the number of tweets published by the hospitals (Figure 3). Although the number of Twitter accounts of private hospitals is greater than public ones, the number of people subscribed to private hospitals has been historically lower than those subscribed to public ones, until January 2015, when the trend reversed. Subscriptions to YouTube are dramatically different between private to public hospitals.

As opposed to our findings regarding Twitter, analysis of the evolution of Facebook profiles (Figure 2) compared with the subscriptions (Figure 4, deep blue lines) demonstrates that the acceptance of private hospitals profiles is greater than public ones. From mid-2011, with a similar number of Facebook accounts, the number of subscriptions for private hospitals is dramatically higher than for public hospitals. Regarding videos (Figure 3), before 2012, public hospitals published more videos than private hospitals. However, since the end of 2011, the number of videos posted by private hospitals has increased more rapidly.

**Figure 5:**
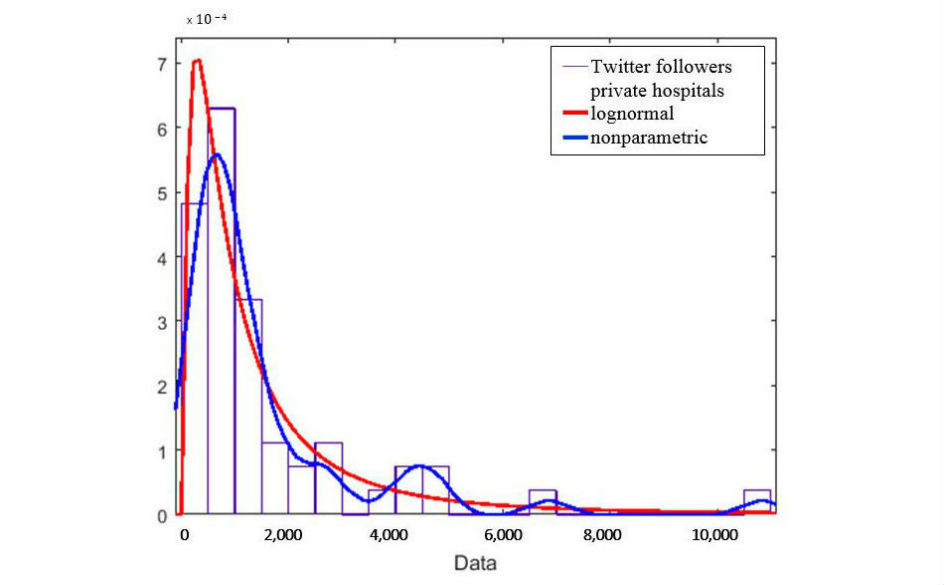
Twitter followers for private hospitals’ distribution (rectangles) fitting comparison between a lognormal distribution (red line) and a nonparametric distribution (blue line).

The position and magnitude attributes of observed variables in Table 1 for each social network separated show a skewed distribution. Kurtosis values (>3) confirm the leptokurtic distribution of all the variables. A similar study in the United States also reported skewed distribution of social media metrics [26]. In this case, statistical analysis was conducted by approximating the skewed distribution to a normal distribution with a lognormal transformation. Our approach is to be as realistic as possible without transforming raw data and using other kinds of statistical tests that can work with nonparametric distributions, such as the Wilcoxon signed-rank test [27]. In Figure 5, we show an example of the raw Twitter follower distribution for private hospitals (histogram) and superimposed the approximation to a normal distribution by applying a lognormal transformation (red line) and a nonparametric distribution (blue line). The goodness of fit or the nonparametric approach is more realistic than the lognormal transformation, and thus, closer to the actual real values of the distribution.

**Table 1:** Magnitude of the observed variables by means of median values, interquartile range, and kurtosis values for public and private hospitals with regard to December 2015.

Variables	Private hospitals			Public hospitals		
	Median	IQR^a^	K	median	IQR	K
Facebook friends (n=266,311)	1253	784.25-2306.75	4.329	740	341.25-2092.25	63.880
Tweets (n=250,040)	1244	641-2780	8.801	750	241-1863	15.565
Twitter followers (n=172,691)	895	502-1576	12.243	437.5	206-973	9.184
YouTube videos (n=4269)	19	5.25-72.25	7.760	19	15.4-62	15.982
YouTube subscribers (n=59,506)	25	4.25-113.5	5.493	12	4-56.75	58.464
YouTube video replays (n=20,488,992)	7386	1530-63,030	14.440	5908	1722.75-38,318.25	50.3166

^a^IQR: interquartile range.

Table 2 shows the results of the independent associations test between public and private hospitals for the observed raw magnitudes, by comparing the nonparametric distribution analysis (two-tailored Wilcoxon test *,* alpha=.05) and the lognormal approximation to a normal distribution (two-tailored *t* student test, alpha=.05).

**Table 2:** Comparative table of the two-sided *t* student test and Wilcoxon test at a 95% CI for private and public hospital comparison on each of the observed variables.

Variables	Lognormal transformation		Nonparametric distribution	
	*P*	CI	*P*	*z*
Facebook friends (n=266,311)	*<.001*^a^	25.277-32.399	.07	1.804
Tweets (n=250,040)	*<.001*	5.728-9.295	*<.001*	2.542
Twitter followers (n=172,691)	*<.001*	4.090-5.220	*<.001*	3.503
YouTube videos (n=4269)	.20	.47	0.709	−0.232 to 1.084
YouTube subscribers (n=59,506)	*.03*	0.048-1.170	.51	0.647
YouTube video replays (n=20,488,992)	*<.001*	4.8474-6.5396	.87	−0.15849

^a^Statistically significant values are given in italics.

## Discussion

### Principal Findings

The Internet presence of Spanish hospitals is high (550/787, 69.9%); nonetheless, the presence on the 3 main social networks is not as high (269/787, 34.2%) when compared with results of previous studies in the United States and Western Europe—which did not include Spain (Table 3).

Hospitals in Spain are adopting different types of social networks and using them to disseminate content. Considering all the hospitals with presence on social media, we find that Twitter had the most profiles, followed by Facebook and YouTube. The comparison of presence, use, and impact dimensions between public and private hospitals shows that private hospitals have a better performance and growth on Facebook and YouTube, whereas the growth on Twitter is similar for both public and private hospitals.

**Table 3:** Comparative table of presence among social media of the outcomes of the study and literature.

Social media presence	United States [26]		Western Europe [23]		Spain	
	2010	2014	2011	2014	2011	2014
	%	%	%	%	%	%
Facebook	18	90	67	-	4	10
Twitter	16	40	40	-	3	12
YouTube	-	-	19	-	3	7

The evolution of the presence, use, and impact of Spanish hospitals on social media is of increasing importance lately. Although the presence of Spanish hospitals on social media is low when compared with other countries, results of the analysis on presence, use, and impact of hospitals profiles on social media show that usage is constantly increasing. Since 2011, the presence of private hospitals on the Internet has grown exponentially. Private hospitals have deeper penetration on Facebook and YouTube than public hospitals, especially in the case of YouTube, where the number of subscribers is three times that of public hospitals. These results contrast with the fact that Spanish citizens prefer public hospitals rather than private [5]. Spanish citizens prefer public hospitals, but, could social media campaigns affect this perception depending of the type of social media? The answer to this question may rely on the nature of each social media and their main type of users. Twitter users look for an easy and quick way to gather information. It may be the case that Spanish social media users prefer tweets from public hospitals due to the high confidence of citizens in public health systems (they are more likely to post health content than advertisements) whereas Facebook and YouTube users look for interesting multimedia content. The results of this study show that despite Spanish citizens having an inferior impression of private hospitals, they achieve better metrics on social media than public hospitals. This might mean that marketing campaigns of private hospitals and the higher investment devoted to generating more attractive multimedia material than public hospitals have a positive impact on users. In fact, *Son Espases* [28] and *Sant Joan de Deu* are two hospitals that have proved that marketing campaigns can be very effective in increasing the presence of hospitals on social media. The case of *Son Espases* hospital is very interesting. This hospital was inaugurated in 2011 with a massive marketing campaign carried out during its construction by a social media company. As a result, when this hospital was inaugurated and included on the NHC, the number of followers was significantly high, accounting for almost 32% (3521) of Twitter subscribers of public hospitals at that time. However, after the inauguration, *Son Espases* ’ activity on social media dropped, most probably because the social media company was no longer contracted. This abrupt change correlated with the large decrease in tweets produced at that time. From Figure 2, it can be seen that after the inauguration, Twitter activity of the public hospital maintained moderate growth, while private hospitals saw an increased slope. This suggests how a good media campaign may affect the visibility of a hospital, but also reveals that these activities need to be sustained for the long term.

Similarly, Facebook and YouTube are social platforms that allow hospitals and users to hold conversations (post and comments) in a very different way from Twitter. A previous study suggests that dialogue between hospitals and users (even patients) on these social networks may be a good source of information regarding service quality [29].

If we look at 2015, only 30.5% (96/314) of public and 36.6% (173/473) of private hospitals are involved in at least one of the observed social networks. This shows that hospital management and marketing teams are not aware of the opportunity that social media provide as an effective communication channel. The ability to respond in real time to users and give information on special situations or health campaigns through social media provides a new method of collecting data and assessing the quality of service, which is faster than traditional phone and onsite surveys.

Statistical analysis of the variables of private and public hospitals observed in the study, including Facebook friends, Twitter followers, number of tweets and YouTube videos, subscribers, and replays was performed in two stages. In the first stage, due to the leptokurtic distribution of all the variables (Table 1), a lognormal transformation to approximate a normal distribution was made, following the approach used by other authors [26]. The two-sided *t* student test with a CI of 95% showed that Facebook friends, Twitter followers, and tweets and YouTube video replays reveal a statistically significant different behavior between private and public hospitals (*P*<.001), whereas YouTube videos and subscribers showed a different, nonsignificant behavior. These findings confirm the results of Griffis and colleagues [26]. In the second stage, instead of transforming data, we used a nonparametric distribution to approximate the probability density function and a two-sided Wilcoxon test with a CI of 95%. This time, results show that only Twitter followers and tweets have a statistically significant different behavior (*P*<.001), whereas the other observed variables had a nonsignificant different behavior. The reason for this difference may be that for the second-stage analysis (nonparametric), raw data was used without performing any transformation, which may have led to bias in the data toward a certain direction. Nonetheless, the two-checked statistical significance of different behaviors in the use of Twitter for private and public hospitals confirms the hypothesis of our study.

This study focuses on analyzing the different trends and behaviors that public and private hospitals have in the use of social media, but the statistical tools used to pursue this analysis are a critical issue. This paper suggests using nonparametric techniques such as Wilcoxon test, used before in other scientific studies [27], which performs a better approximation to real data than the lognormal approximation, but the authors cannot confirm this extent.

The research question analyzed in this study is relevant in the era of social communication. Hospitals should have a strong presence on social media just as other entities and corporations do, as they provide an extraordinary and innovative instantaneous communication channel that reaches a wide audience. Unlike traditional communication campaigns, social media allow the release of information in new media formats (infographics, hashtags, audio, and video), adding formal and informal messages (eg, use of smileys) in very short time periods. Another singular characteristic is that hospitals can instantly assess the impact that the message or communication campaign has regarding new subscribers, retweets, or likes. Our study confirms that the performance on Twitter is different between private and public hospitals (Table 2), whereas at the same time, we observe a close evolution in the number of subscribers for both public and private hospitals (Figure 4). The cause may depend on the type of subscribers (age, profession, or interests) and in the type of broadcasted content (promotion, advertisement, or awareness).

When analyzing the performance of hospitals on social media, the risks as well as the ethical aspects of the use of social media in health care should also be considered. It is an important issue that is beyond the scope of our study. Scientific literature has addressed these issues in recent years [30], and several health care and health professional organizations have defined rules and policies on the use of social media. A good example is the American Medical Association (United States) policy, and more specifically, the Use and Style Guidelines on Social Networks published by the Regional Healthcare Agency of Andalucia (Spain) [31]. In spite of the multiple benefits of social media, there are significant risks that should be taken into account, mainly concerning security and privacy of the users. Broadcasters (hospitals) and consumers (patients and citizens) are not usually aware of the size of the audience they reach when a comment or an opinion is posted on any of the social media. The convenience of communicating with digital friends may lead users to publish harmful or inappropriate material that may affect their reputation and which may be very difficult (not to say impossible) to erase. Therefore, the use of social media by hospitals should adhere to a high level of compliance to published guidelines and rules from relevant organizations.

### Limitations

Our findings are based on the entire population of public and private hospitals in Spain; nonetheless, even though the data collection method was based on previous publications, the authors cannot guarantee the location of all the webpages and social media profiles of the hospitals in the NHC. The fact that social media are constantly and rapidly changing affects the way data are collected and analyzed. Finally, some hospitals are outliers in the way that their performance shows a comparatively higher use and impact on social media than others, regardless of being public or private, and this could need further analysis, which is out of the scope of this study.

### Future Work

Future work will tackle analysis of the subscribers’ public profile and each profile on each social media, and among them, which type of subscribers are more likely to interact with posted content (likes, comments, shares) and which type of information has greater or lesser impact.

### Conclusions

The presence of Spanish hospitals on social media is constantly evolving, showing an incremental growth year by year; however, it is very low compared with hospitals in the United States and Western Europe. Public hospitals are more active on Twitter, whereas private hospitals have a better performance on Facebook and YouTube. The Spanish health care system needs to maintain a high-ranking position, and to do so, this study suggests that hospitals, both public and private, should devote more effort to and be more aware of social media. The study conclusion is that private hospitals and public hospitals show statistically significant different behaviors in their use of Twitter (number of tweets and number of followers).

## Abbreviations

FB: Facebook
IQR: interquartile range
NHC: National Hospitals Catalog
TW: Twitter
YT: YouTube
